# Interleukin-10 Attenuates Liver Fibrosis Exacerbated by Thermoneutrality

**DOI:** 10.3389/fmed.2021.672658

**Published:** 2021-05-26

**Authors:** Ha Thi Nga, Ji Sun Moon, Jingwen Tian, Ho Yeop Lee, Seok-Hwan Kim, Young-Sun Lee, Jae-Han Jeon, Hyon-Seung Yi

**Affiliations:** ^1^Laboratory of Endocrinology and Immune System, Chungnam National University School of Medicine, Daejeon, South Korea; ^2^Department of Medical Science, Chungnam National University School of Medicine, Daejeon, South Korea; ^3^Research Center for Endocrine and Metabolic Diseases, Chungnam National University School of Medicine, Daejeon, South Korea; ^4^Department of Surgery, Chungnam National University School of Medicine, Daejeon, South Korea; ^5^Department of Internal Medicine, Korea University College of Medicine, Seoul, South Korea; ^6^Department of Internal Medicine, School of Medicine, Kyungpook National University, Daegu, South Korea

**Keywords:** liver fibrosis, hepatic stellate cells, thermoneutrality, brown adipose tissue, IL-10, conditioned media

## Abstract

**Background:** Crosstalk between brown adipose tissue (BAT) and the liver is receiving increasing attention. This study investigated the effect of BAT dysfunction by thermoneutral (TN) housing on liver fibrosis in mice and examined the effect of secreted factors from brown adipocytes on the activation of hepatic stellate cells (HSCs).

**Methods:** The carbon tetrachloride (CCl_4_)-induced liver fibrosis mouse model was used to evaluate fibrotic changes in the livers of mice housed under standard and TN conditions. The effect of BAT on the activation of HSCs was examined using cultured cells treated with conditioned media from brown adipocytes.

**Results:** Under TN conditions, mice with CCl_4_-induced liver fibrosis exhibited increased liver injury, collagen deposition, and alpha smooth muscle actin (α-SMA) expression in the liver compared with mice maintained at room temperature. The numbers of liver-infiltrating immune cells and T cells producing IL-17A and IFN-γ were also significantly increased in the livers of mice housed under TN conditions. Treatment of HSCs with conditioned media from brown adipocytes markedly attenuated HSC activation, as shown by down-regulated α-SMA expression at day 4, day 7 and day 10 of culture. At thermoneutrality, with CCl_4_ administration, IL-10-deficient mice exhibited more severe liver fibrosis than wild-type mice. Interestingly, conditioned media from IL-10-deficient brown adipocytes could up-regulate the expression of α-SMA and induce HSCs activation.

**Conclusions:** BAT inactivation by thermoneutrality contributes to the activation of pro-inflammatory and pro-fibrotic pathways in mice with CCl_4_-induced liver fibrosis. Normal brown adipocytes secreted factors that impair the activation of HSCs, while this protective effect was lost in IL-10-deficient brown adipocytes. Thus, the BAT–liver axis may serve as a potential therapeutic target for liver fibrosis, and IL-10 may be a key factor regulating the activation of HSCs by BAT.

## Introduction

Fibrosis is a marker of chronic liver disease and a major risk factor for the development of hepatocellular carcinoma (1, 2). A previous study reported that the degree of liver fibrosis positively correlated with the mortality rate in non-alcoholic fatty liver disease (NAFLD) patients (3). A key event in the initiation of hepatic fibrosis is the activation of hepatic stellate cells (HSCs) (2, 4). Upon liver injury, HSCs transdifferentiate into myofibroblasts and become the major producers of hepatic collagen (4). Primary HSCs derived from normal mouse livers are in a quiescent state, and when these cells are plated on plastic tissue culture plates, they lose their storage of retinoids and begin to express pro-fibrotic markers such as α-smooth muscle actin (α-SMA) (4). A previous review summarized several cytokines involved in HSC activation, including platelet-derived growth factor (PDGF), transforming growth factor-β (TGF-β), interferon-γ (IFN-γ), and interleukins such as IL-10, IL-6, IL-1, IL-17, and IL-22 (1). More interestingly, in the liver, IL-1 and IL-17 induce the activation of HSCs, while IL-10 and IL-22 have been shown to be anti-fibrogenic (1, 5).

Thermoneutrality refers to the temperature at which the metabolic rate (energy expenditure) required to maintain body temperature is the lowest (6). For healthy mice, thermoneutral (TN) temperature is generally 30–32°C (6). Experimental mice are generally housed at room temperature (20–23°C). Under these conditions, C57BL/6J mice exhibit mild cold stress, with an ~2-fold increase in energy expenditure compared with those housed at 30°C (7). In addition to these metabolic effects, a previous report demonstrated that cold stress associated with standard housing can impair immune responses (8), while mice housed under TN conditions show increased immune responses (9). TN housing accelerates the pathogenesis of high-fat diet-induced NAFLD, as demonstrated by increased hepatic triglyceride (TG) levels, lipid accumulation, infiltrating CD11b+F4/80+ immune cells, and the expression of genes related to lipid mediators, collagen formation, and apoptotic signaling (10).

Brown adipose tissue (BAT) is a key thermogenic organ that protects against cold and maintains the core body temperature through non-shivering thermogenesis (11, 12). This function is carried out by uncoupling protein (Ucp1), which is exclusively expressed in the inner membrane of the mitochondria of brown adipocytes (13). Dysfunctional BAT is observed in many types of metabolic disorders (11, 14). Metabolic disorders are associated with hepatic fat deposition, endoplasmic reticulum stress, lipotoxicity, and parenchymal cell injury and death in the liver (15). These factors can induce hepatic inflammation, HSC activation, and progressive fibrogenesis. Besides its heat-producing capacity, BAT also acts as a secretory organ that regulates whole body metabolism through endocrine factors (11, 16). Recently, an increasing number of studies have suggested a link between BAT and the liver (16–18). Neuregulin (NRG4), a member of the epidermal growth factor family that is highly expressed in the BAT, can bind selectively to hepatocytes and regulate liver lipogenesis (16). Another study demonstrated that BAT can inhibit liver steatosis by clearing circulating free fatty acids (FFAs) and blocking lipid trafficking into the liver (18). This study also revealed that activated brown adipocytes can secrete adiponectin and IL-6 to inhibit primary hepatocyte death (18).

As a well-known anti-inflammatory cytokine, IL-10 can activate signal transducer and activator of transcription 3 (STAT3) in Kuppfer cells and regulate liver inflammation (19). A high-fat diet or ethanol feeding can increase liver inflammation in IL-10-deficient mice (20). Intriguingly, IL-10 knock-out mice also display less steatosis and lower levels of serum alanine aminotransferase (ALT) (20). IL-10 can also block the production of IL-6, impairing liver regeneration and increasing liver damage (19). However, this cytokine can also inhibit pro-inflammatory responses stimulated by tumor necrosis factor-α (TNF-α) or lipopolysaccharide (LPS), thereby ameliorating liver injury (19). Notably, a recent study indicated that IL-10 is associated with BAT function (21). By quantification of oxygen consumption rates in isolated mitochondria, José C. de-Lima-Júnior et al. reported that the BAT mitochondria of IL-10-deficient mice had impaired Ucp1-dependent respiration (21). Furthermore, these mice had a lower BAT temperature and the BAT mitochondria showed structural damage. IL-10 knock-out mice exposed to cold also showed impaired thermogenic capacity (21). We therefore hypothesize that inadequate BAT activity under TN conditions and deletion of IL-10 may accelerate hepatic fibrosis progression.

## Methods

### Animals and Liver Fibrosis Induction

Male C57BL/6N wild-type (WT) or IL-10 knock-out (IL-10 KO) mice (10–11 weeks old) were used. IL-10 knock-out mice were kindly provided by Prof. Jae-Han Jeon (Kyungpook National University, Daegu, Korea). All animals were housed in groups of 3–5 in a 12 h light/dark cycle under standard conditions and provided with food and water ad libitum. Mice were allowed to acclimatize for about 1 week and randomly assigned to two housing conditions: ambient temperature (22 ± 2°C) or thermoneutrality (30°C). Animals in the TN group were housed in a chamber stably set at 30°C. In age-matched groups of mice, liver fibrosis was induced by three intraperitoneal injections per week of CCl_4_ (2 ml/kg body weight, diluted 1:9 in olive oil) for 3 weeks. The body weight was measured before each injection. Mice were sacrificed 12 h after the last CCl_4_ injection. Blood glucose was measured using a glucometer before sacrifice. The liver and BAT were removed, weighed, snap-frozen in liquid nitrogen, and stored at −80°C or fixed in 10% neutral formalin for further analysis. All experimental procedures were conducted in accordance with the guidelines of the Institutional Animal Care and Use Committee of Chungnam National University School of Medicine (CNUH-019-A0071, Daejeon, Korea).

### Liver Mononuclear Cell (MNC) Isolation and Flow Cytometry

Liver mononuclear cells (MNCs) were isolated as described previously (22). Specifically, liver tissues were collected and washed in cold phosphate-buffered saline (PBS). Immediately, the tissues were minced and the pieces were incubated in DMEM containing dissociation enzymes (collagenase I) at 37°C for 30 min with shaking (120 rpm). After that, the tissue was homogenized on a GentleMACS Dissociator (Miltenyi Biotec, Bergisch Gladbach, Germany) using the program m_liver_03.01. Mouse liver tissues were passed through a cell strainer with a 70 μm nylon mesh filter (BD Falcon, Millville, NJ, USA) to remove debris, and the liver cell suspensions were then centrifuged in cold PBS at 500 rpm for 5 min to eliminate hepatocytes. The supernatant containing the MNCs was collected and centrifuged at 1,600 rpm at 4°C for 10 min. The cell pellet was collected, resuspended in 40% Percoll (Sigma-Aldrich, St. Louis, MO, USA) in PBS, and then centrifuged at 2,400 rpm at 4°C for 30 min without the brake. The supernatant was removed and red blood cells were removed by treatment with an RBC lysis buffer for 2–5 min, depending on the number of cells. The samples were then centrifuged at 1,800 rpm at 4°C for 10 min. Finally, MNCs were resuspended in RPMI 1,640, counted, and prepared for flow cytometry.

Isolated liver MNCs were washed in FACS buffer [Dulbecco's PBS (DPBS) containing 0.5% bovine serum albumin (BSA) and 0.05% sodium azide] and then stained with fluorochrome-conjugated anti-CD45, anti-CD44, anti-CD62L, anti-CD3, anti-CD4, anti-CD8, anti-Foxp3, anti-CD11b, anti-Ly6G, anti-Ly6C, and anti-CD25 antibodies (eBioscience/Thermo Fisher Scientific, Waltham, MA, USA). For detection of regulatory T cells (Treg), anti-Foxp3 was added after cell permeabilization. For intracellular staining, PE-conjugated anti-IFN-γ and anti-IL17A antibodies (BD Biosciences, San Jose, CA, USA) were used. Intracellular cytokine staining was performed after re-stimulation of cells with phorbolmyristate acetate/ionomycin/brefeldin A for 5 h using the BD Cytofix/Cytoperm kit (BD Biosciences). The stained cells were collected on a BD LSR II Flow Cytometer (BD Biosciences) and analyzed using FlowJo software (Tree Star, Ashland, OR).

### Serum Biochemistry Measurements

Blood samples were taken from the facial vein and allowed to clot for 30 min at room temperature. Serum was collected after centrifugation at 10,000 rpm for 10 min at 4 °C and stored at −80 °C until use. The serum levels of alanine aminotransferase (ALT), aspartate aminotransferase (AST), total cholesterol (TCHO), and triglycerides (TGs) were measured using a Fuji Dri-Chem 4,000i analyzer (Fujifilm, Tokyo, Japan). IL-6 and IL-10 serum levels were measured using an enzyme linked immunosorbent assay (ELISA) kit (R&D Systems, Minneapolis, MN, USA).

### Histology

Liver and BAT sections were fixed in 10% neutral buffered formalin at room temperature and embedded in paraffin. After deparaffinization in xylene and rehydration with ethanol, 4 μm-thick sections were stained with hematoxylin and eosin (H&E) and 0.1% Sirius Red (Sigma-Aldrich) for detection of collagen deposition in the liver. For immunohistochemistry for α-SMA, staining was performed by incubation with an α-SMA antibody overnight at 4°C, followed by detection using 3, 3'-diaminobenzidine (DAB) as the chromogen/substrate.

### Isolation of Mouse Primary Hepatic Stellate Cells

HSCs were isolated as described previously (22). Briefly, mice were perfused *in situ* through the portal vein with 1x EGTA [5.4 mM KCl, 0.44 mM KH_2_PO_4_, 140 mM NaCl, 0.34 mM Na_2_HPO_4_, 0.5 mM EGTA, 25 mM Tricine, and 1% penicillin/streptomycin (PS); pH 7.2] at a rate of 1.4 ml/min, followed by perfusion with 0.075% collagenase type I in HBSS at 37°C. Liver tissues were collected into digestion solution and homogenized. The tissue was incubated for 20 min at 37°C with shaking (100 rpm). The cell suspension was filtered through a 70 μm nylon cell strainer (BD Falcon) and centrifuged at 500 rpm for 5 min at room temperature to separate the hepatocytes. The supernatant was collected and centrifuged at 1,600 rpm at 4°C for 10 min, and the cell pellet was collected, resuspended in HBSS, and centrifuged again. Gradient solutions were prepared with Opti-Prep (40, 20, and 11.5%). The pellet was resuspended in 20% Opti-Prep (Sigma-Aldrich), slowly overlaid with 11.5% Opti-Prep and 0% Opti-Prep (HBSS), and centrifuged at 3,000 rpm at 4°C for 17 min without the brake. HSCs were collected from the interface of the 0 and 11.5% Opti-Prep layers. Freshly isolated primary HSCs were washed with HBSS, resuspended in media [RPMI 1,640 medium with 10% fetal bovine serum (FBS) and 1% antibiotics], and counted.

### Primary Brown Adipocyte Isolation and Differentiation

BAT was dissected from 3-week-old male mice, washed in 1x PBS, and minced in isolation buffer (0.123 M NaCl, 5 mM KCl, 1.3 mM CaCl_2_, 5 mM glucose, 100 mM HEPES, 4% BSA, and 1 mg/ml collagenase type 2). The tissue was incubated for 40 min at 37°C with shaking at 100 rpm. The digested tissue was filtered through a 70 μm nylon cell strainer and centrifuged at 1,300 rpm for 5 min. The pellet consisting of precursor cells was washed twice with culture media (DMEM containing 15% FBS and 1% PS). The pellet was resuspended in 10 ml of culture media and cells were seeded on 100 mm dishes or in 6-well plates.

Brown adipocytes were grown to confluence in culture medium. Confluent cells were incubated for an additional 24 h. The culture media was removed and replaced with differentiation media containing 0.5 mM 3-isobutyl-1-methylxanthine (IBMX), 0.5 μM dexamethasone, 20 nM insulin, 0.125 mM indomethacin, and 1 nM T3. After 2 days, the cells were maintained in media containing 20 nM insulin and 1 nm T3 for 4–5 days. Cell culture media was collected from brown adipocytes when they exhibited a fully differentiated phenotype, with accumulation of multilocular fat droplets.

Differentiated brown adipocytes were maintained in DMEM supplemented with 10% FBS serum and 1% PS in a humidified incubator containing 5% CO_2_. When cells reached 80% confluence, they were cultured for 24 h with fresh media. Conditioned media were collected after 24 h culture of 80% confluent of brown adipocytes or 100% differentiated brown adipocytes. Cell supernatants were collected, filtered, aliquoted, and stored for further analysis.

### Protein Preparation and Western Blotting

Tissues and cells were homogenized and lysed in RIPA lysis buffer containing protease and phosphatase inhibitors. The supernatants were collected after centrifugation at 13,000 rpm for 15 min at 4°C. Protein concentrations were measured, and protein samples were separated by 10% sodium dodecyl sulfate (SDS)-polyacrylamide gel electrophoresis. After that, the proteins were transferred onto nitrocellulose or polyvinylidene fluoride (PVDF) membranes. Non-specific binding sites were blocked by incubating the membranes in 5% skim milk for 1 h. The blots were incubated with primary antibodies in Tris-buffered saline with Tween (TBST) containing 0.5% skim milk overnight at 4°C. The membranes were then incubated with horseradish peroxidase (HRP)-conjugated secondary antibodies for 2 h at room temperature and the proteins were detected by enhanced chemiluminescence (ECL). Membranes were visualized on an ODYSSEY instrument and bands were quantitated using Image Studio Software (LI-COR Biosciences, Lincoln, NE, USA).

### Total RNA and Real-Time PCR

Total RNA was isolated from cells or tissues using TRIzol reagent (Thermo Fisher Scientific) according to the manufacturer's protocol. Complementary DNA (cDNA) was reverse-transcribed from the same quantity of total RNA using oligo-dT primers and M-MLV reverse transcriptase (Invitrogen/Thermo Fisher Scientific). Real-time PCR was performed with SYBR Green Real-Time PCR Master Mix on a 7,500 Fast Real-Time PCR system (Applied Biosystems, Carlsbad, CA). The comparative Ct method was used to quantify transcript levels, which were normalized against the level of 18S mRNA.

### Statistical Analysis

Statistical analyses were performed using GraphPad Prism 8 software (GraphPad, San Diego, CA, USA). To compare values from two groups, Student's *t-*tests were performed. A *p*-value < 0.05 was considered statistically significant.

## Results

### TN Housing Accelerates Brown Adipose Dysfunction in Mice With Liver Fibrosis

**Figure 1:**
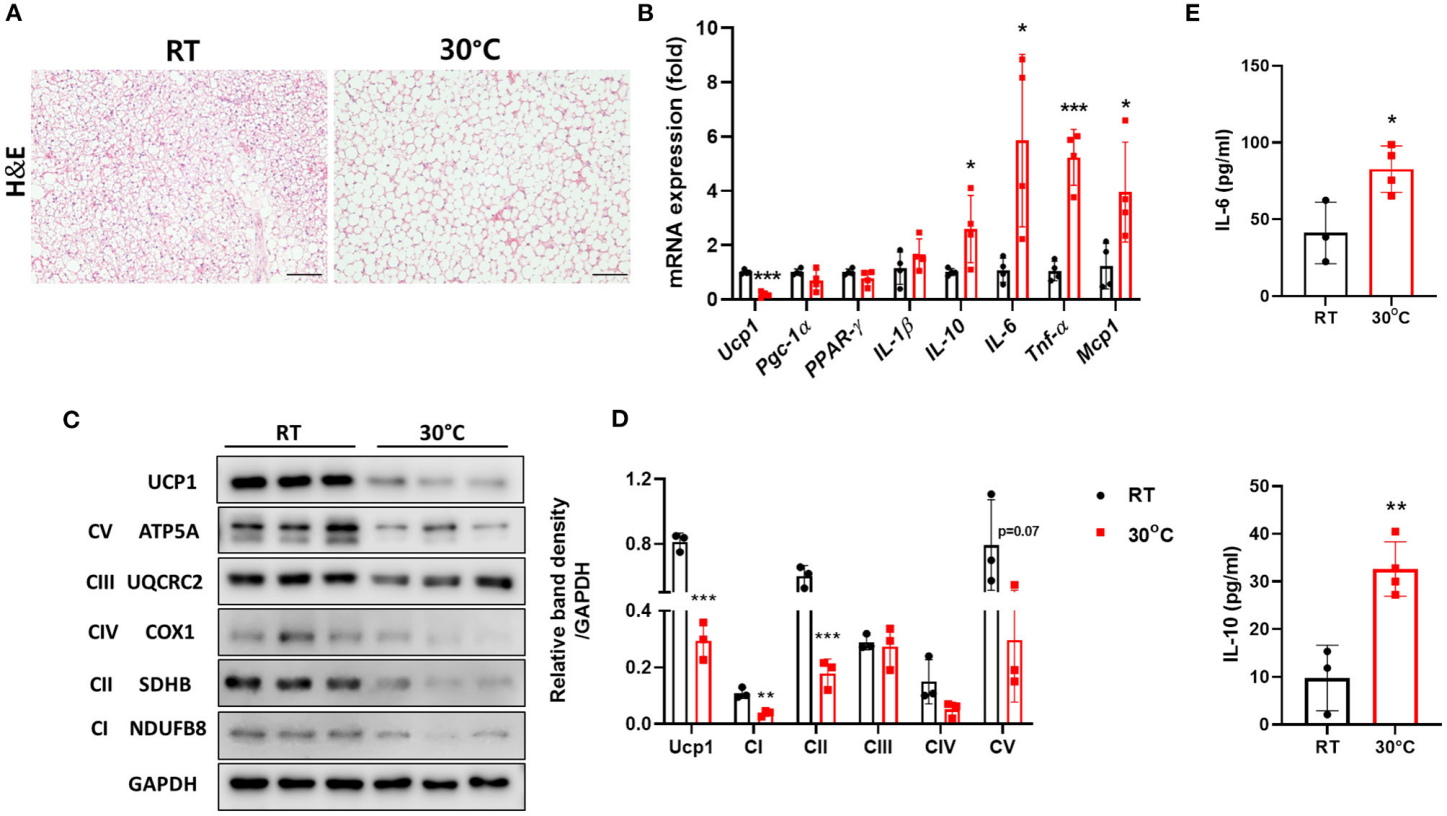
Thermoneutral conditions impaired BAT function in mice with hepatic fibrosis. Mice with CCl_4_-induced fibrosis were housed at room temperature or thermoneutrality. **(A)** H&E staining of BAT (original magnification, × 20). **(B)** Whole BAT tissues were subjected to real-time PCR for inflammatory genes. **(C)** Whole BAT tissues were subjected to western blotting for proteins involved in mitochondrial function. **(D)** Relative band density of OXPHOS protein expression. **(E)** Serum IL-6 and IL-10 were measured by ELISA. Statistically significant differences were determined by Student's *t-*tests. *p < 0.05, **p < 0.01, ***p < 0.001.

It has been suggested that housing temperature impacts BAT and its activity is decreased at thermoneutrality (23). We housed mice with CCl_4_-induced liver fibrosis under TN conditions to impair BAT function as well as to characterize the effect of TN housing on hepatic fibrosis. As expected, the structure of the BAT was abnormal in mice housed under TN conditions, as indicated by the increased size of lipid droplets (Figure 1A). BAT is rich in mitochondrial Ucp1, which plays an important role in thermogenesis (13). TN conditions significantly decreased Ucp1 expression both at transcriptional and protein levels (Figures 1B,C). Compared with room temperature housing, TN housing also increased the expression of pro-inflammatory cytokines, including *IL-6, TNF-*α, and *MCP-1* in the BAT (Figure 1B). The expression of the mitochondrial oxidative phosphorylation (OXPHOS) complex was also down-regulated in the BAT of mice with liver fibrosis housed under TN conditions (Figures 1C,D). Thus, TN conditions impaired the function of BAT by reducing both Ucp1 levels and mitochondrial oxidative capacity. The serum levels of IL-6 and IL-10 were also significantly increased in mice housed at 30°C compared with mice housed under standard conditions (Figure 1E). These data suggest that TN housing can impair BAT function and induce a severe systemic inflammatory response in mice with CCl_4_-induced liver fibrosis. Moreover, under TN conditions, the inflamed BAT may be a source of cytokines that enhance systemic inflammation.

### TN Housing Exacerbates Hepatic Fibrosis in the CCl_4_-Induced Fibrosis Model

**Figure 2:**
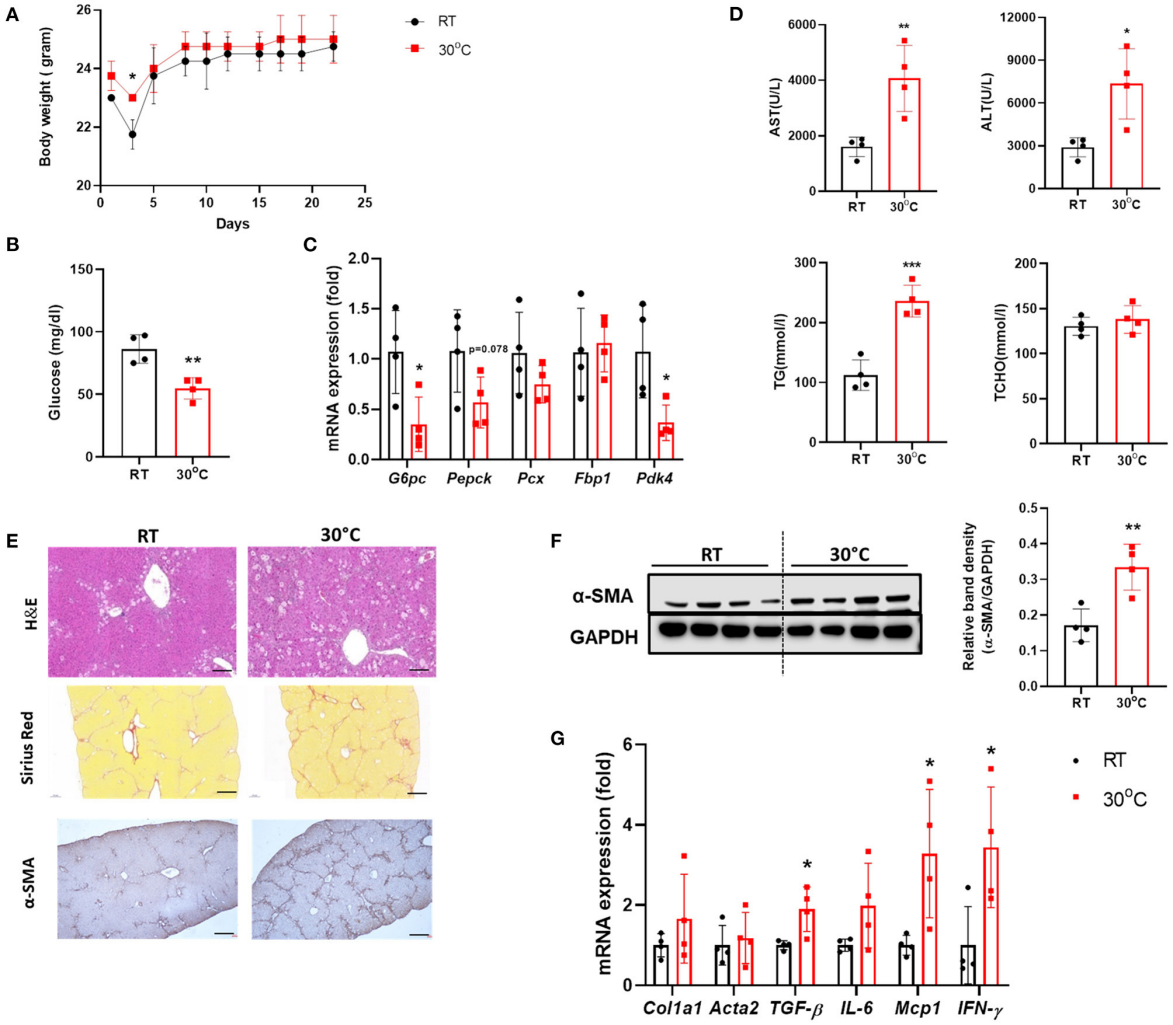
Thermoneutral housing exacerbated liver fibrosis in mice. Mice with hepatic fibrosis induced by CCl_4_ injection were housed under TN or standard conditions. **(A)** The body weight was measured before each injection. **(B)** The fasting blood glucose was measured at the time of sacrifice. **(C)** Whole liver tissues were subjected to real-time PCR. **(D)** Serum AST, ALT, TG, and TCHO levels were assessed. **(E)** Liver sections were stained with H&E (original magnification, × 20), Sirius Red (original magnification, × 4), or an α-SMA antibody (original magnification, × 4). **(F)** Whole liver tissues were used for western blotting to detect α-SMA. **(G)** Whole liver tissues were subjected to real-time PCR. Statistically significant differences were determined by Student's *t-*tests. *p < 0.05, **p < 0.01, ***p < 0.001.

To investigate the effect of BAT dysfunction on liver fibrosis, we examined the livers of mice with CCl_4_-induced hepatic fibrosis housed under TN conditions. There was no significant difference in body weight between the two groups of mice after 3 weeks of CCl_4_ administration (Figure 2A). At the time of sacrifice, the fasting blood glucose levels were measured. Mice in the TN group had lower levels of fasting glucose compared with controls housed at a standard temperature (Figure 2B). In the fasted state, hepatic gluconeogenesis is the primary source of glucose production (24). The expression of genes related to gluconeogenesis in the liver was also down-regulated in CCl_4_-injected mice housed under thermoneutrality (Figure 2C). Importantly, TN conditions also increased the severity of liver injury, as indicated by significantly higher serum levels of AST, ALT, and TGs in the TN group, without any change in the TCHO level (Figure 2D). H&E staining showed a large population of infiltrating immune cells in the livers of mice adapted to 30°C (Figure 2E). Mice in this group also showed an increase in the expression of fibrosis markers, including collagen and α-SMA. These findings were confirmed by Sirius Red staining and immunohistochemistry and western blotting for α-SMA (Figures 2E,F). Moreover, the expression of the pro-inflammatory cytokines *IL-6* and *MCP-1* and the fibrotic marker *TGF-*β were also markedly elevated in the livers of mice under TN conditions by real-time PCR (Figure 2G). Collectively, these findings strongly suggest that TN housing can aggravate CCl_4_-induced liver fibrosis in mice.

### TN Housing Enhances Immune Infiltration of the Liver

**Figure 3:**
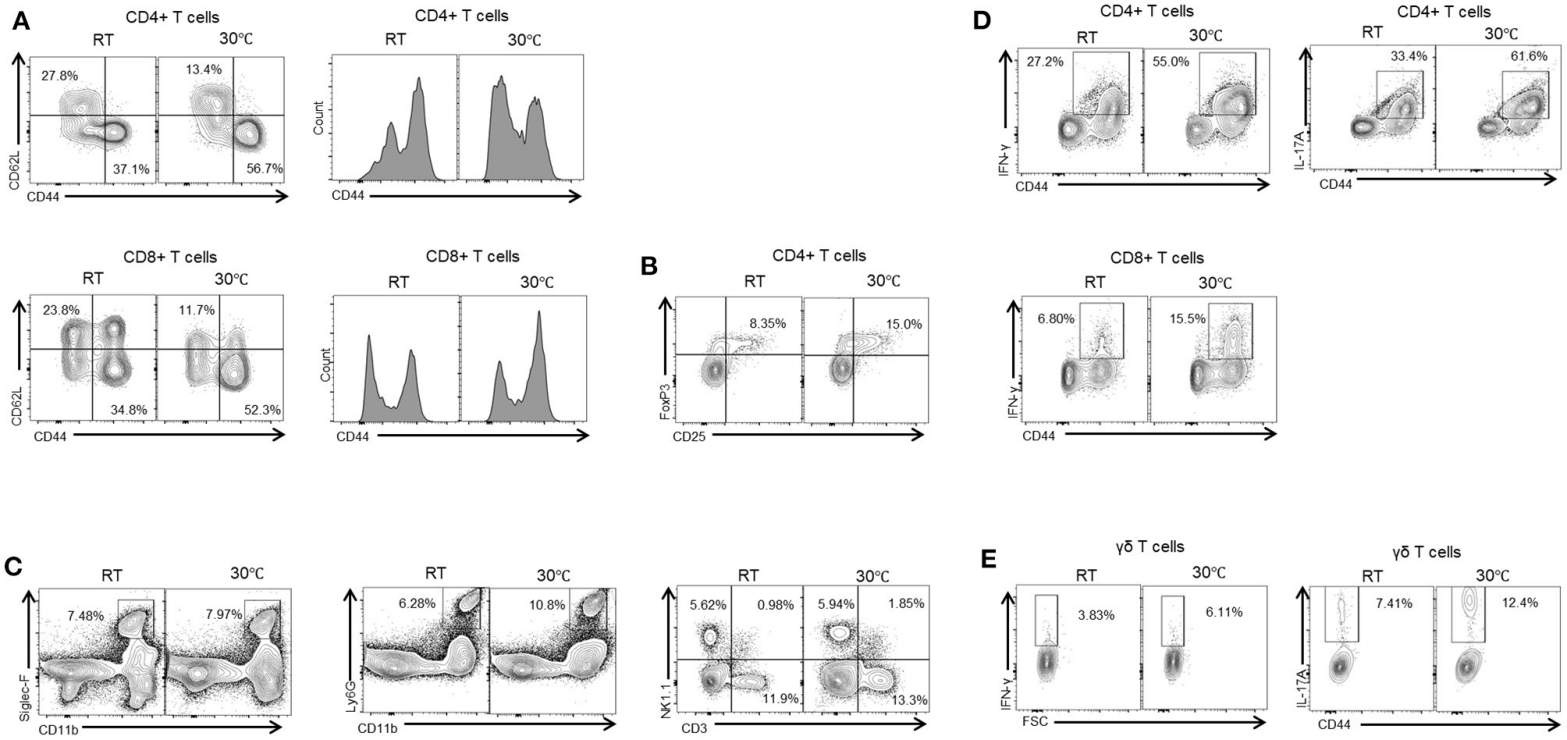
Thermoneutral housing enhances immune cells infiltration of the liver. Isolated liver MNCs were analyzed for CD4+ T cells, CD8+ T cells **(A)**, regulatory T cells **(B)**, monocytes, neutrophils, and NK cells **(C)** by flow cytometry. Liver MNCs were evaluated through intracellular staining for IFN-γ-producing and IL-17A-producing CD4+ T cells, CD8+ T cells **(D)**, and γδ T cells **(E)**.

Liver fibrosis is a result of complex interactions among various types of liver cells. A number of studies have demonstrated the important role of the immune response in fibrosis progression (4, 25). Liver immune cells include infiltrating T cells, macrophages, neutrophils, and Kupffer cells, all of which can contribute to hepatic inflammation. Hepatic inflammation can induce the activation of HSCs and lead to the generation of myofibroblasts (4). We therefore characterized the resident liver immune cells in mice housed under standard or TN conditions. Compared with the TN group, the mice with standard housing showed a higher proportion of naïve CD4+ and CD8+ T cells (CD62L+CD44-) in the liver. By contrast, there was an increase in activated CD4+ and CD8+ T cells (CD62L-CD44+) in the livers of mice in the TN group (Figure 3A). In addition, mice in the TN group displayed increased hepatic immune infiltration of other immune cell types, including Treg (Figure 3B), eosinophils (Siglec-F+CD11b+), neutrophils (CD11b+Ly6G^high^), natural killer (NK) cells (CD3-NK1.1+), natural killer T (NKT) cells (CD3+NK1.1+), and total T cells (CD3+NK1.1-) (Figure 3C) compared with control mice. Furthermore, through intracellular staining, mice housed under TN conditions were observed to have increased numbers of IL-17A- and IFN-γ-positive CD4+ and CD8+ T cells (Figure 3D) and gamma delta (γδ) T cells (Figure 3E). Notably, previous studies have suggested the critical role of IL-17A and IFN-γ in the pathogenesis of hepatic fibrosis (26, 27). Zhongming Tan et al. demonstrated that IL-17A induced IL-6, IL-1β, and TNF-α expression, leading to liver inflammation and fibrosis in the CCl_4_ model. Furthermore, the authors showed that IL-17A activated HSCs via the ERK1/2 and p38 mitogen-activated protein kinase (MAPK) pathways (26). Another study demonstrated that hepatic γδ T cells produced high levels of IL-17 and IFN-γ during chronic liver injury, and IFN-γ was shown to exert a protective effect during fibrogenesis (27). In agreement with this previous data, we found that thermoneutrality increased the infiltration of the liver by immune cells and increased the secretion of inflammatory cytokines in mice with liver fibrosis.

### Conditioned Media From Brown Adipocytes Inhibited the Activation of HSCs

**Figure 4:**
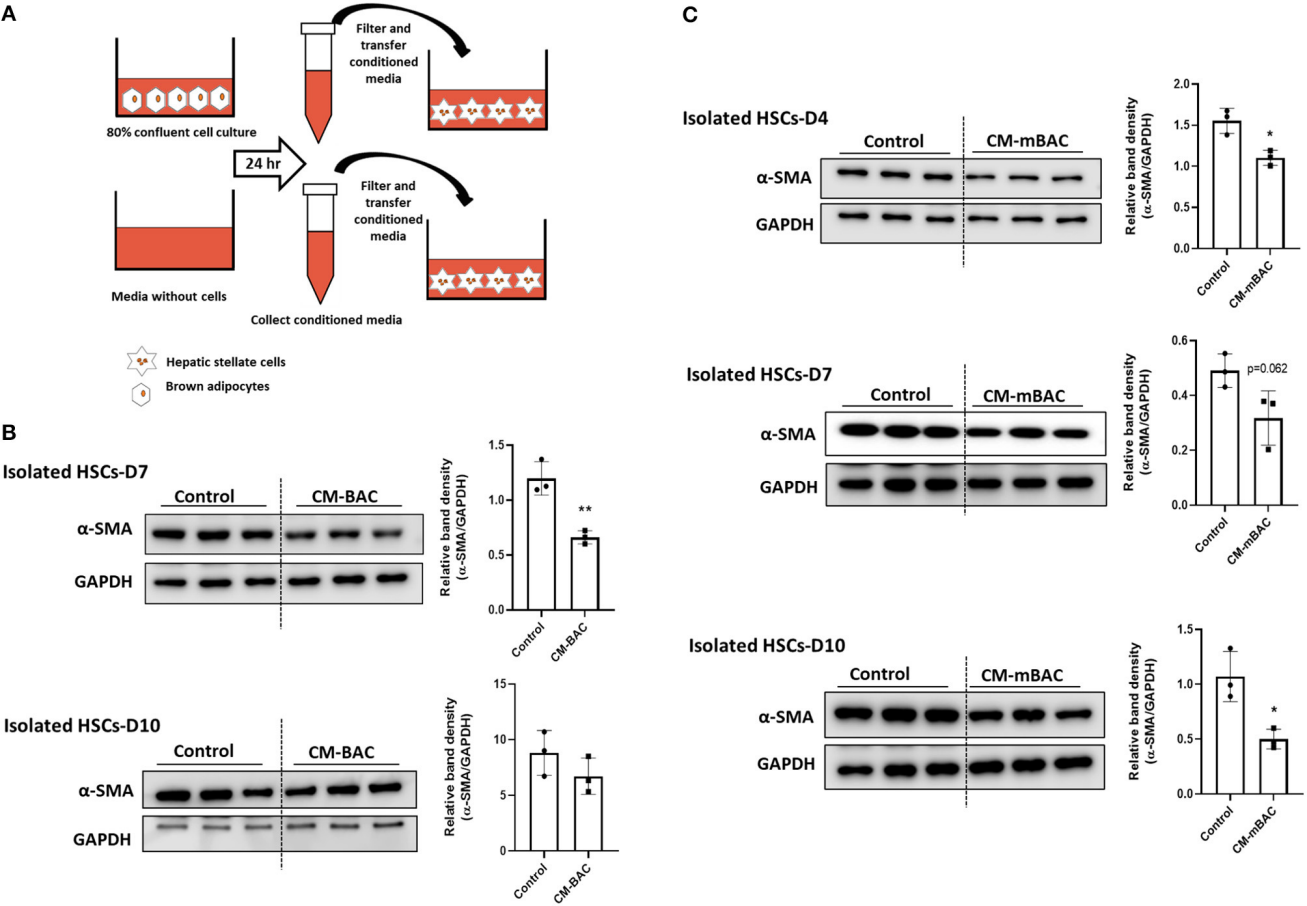
Conditioned media from brown adipocytes inhibited HSC activation. HSCs were isolated from WT mice. At day 4, day 7, and day 10, HSCs were cultured with conditioned media from brown adipocytes (CM-BAC) or differentiated brown adipocytes (CM- mBAC) for 24 h. CM-BAC was collected after 24 h from brown adipocytes grown to 70–80% confluence. CM-mBAC was collected after culture of fully differentiated brown adipocytes for 24 h. **(A)** Schematic description of HSCs culture with CM-BAC. After 24 h of culture with CM-BAC **(B)** or CM-mBAC **(C)** HSCs were collected and subjected to western blotting for α-SMA. Statistically significant differences were determined by Student's *t-*tests. *p < 0.05, **p < 0.01.

HSCs play the principal role in the synthesis of collagen during liver fibrosis process (4). HSC activation, as indicated by α-SMA expression, is a crucial marker of hepatic fibrosis. To examine the function of BAT on liver fibrosis, primary HSCs isolated from WT mice were cultured with conditioned media derived from brown adipocytes. Compared with control media, conditioned media from brown adipocytes (CM-BAC) impaired the activation of primary HSCs by reducing the expression of α-SMA at day 7 and day 10 of culture (Figure 4A). Conditioned media from differentiated brown adipocytes (CM-mBAC) also significantly inhibited the expression of α-SMA in HSCs at day 4, day 7, and day 10 (Figure 4B). Therefore, both brown adipocytes and differentiated brown adipocytes can regulate HSC activation, and potentially liver fibrosis, through secreted factors. Thus, we propose that at room temperature, BAT maintains the function of HSCs and inhibits their activation, while under TN conditions, the inflamed BAT loses its function and is unable to inhibit HSC activation.

### IL-10 KO Mice Exhibit Severe CCl_4_-Induced Fibrosis at Thermoneutrality

**Figure 5:**
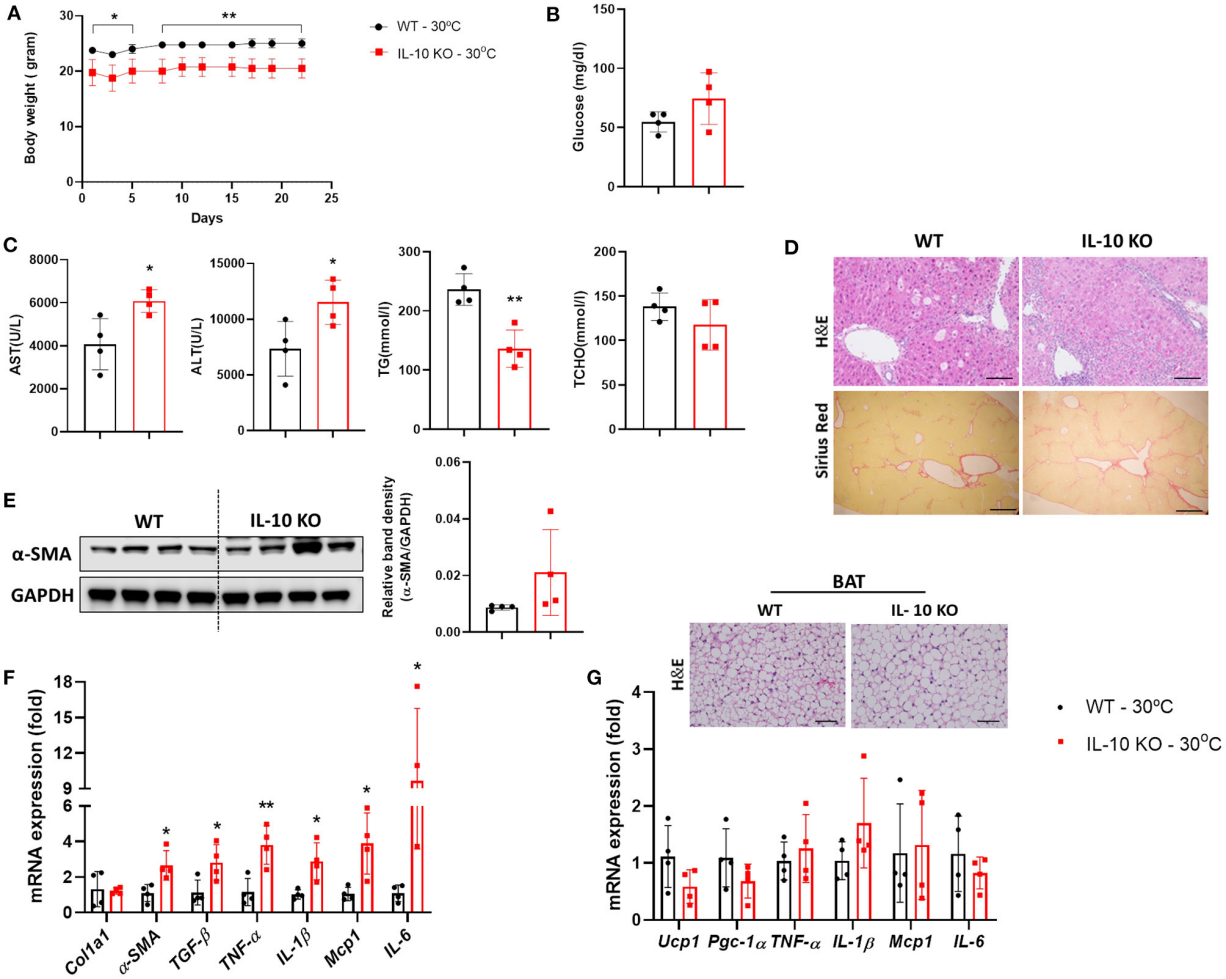
IL-10-deficient mice displayed severe liver fibrosis under thermoneutral conditions. Mice with hepatic fibrosis induced by CCl_4_ injection were housed under TN or standard conditions. **(A)** The body weight was determined before each injection. **(B)** The fasting blood glucose was measured at the time of sacrifice. **(C)** Serum AST, ALT, TG, and TCHO levels were assessed. **(D)** Whole liver tissues were used for western blotting to detect α-SMA. **(E)** Liver sections were stained with H&E (original magnification, × 20) and Sirius Red (original magnification, × 4). **(F)** Whole liver tissues were analyzed by real-time PCR. **(G)** Whole BAT tissues were subjected to real-time PCR for detecting inflammatory genes and BAT sections were stained with H&E (original magnification, × 10). Statistically significant differences were determined by Student's *t-*tests. *p < 0.05, **p < 0.01.

Notably, serum levels of IL-10 were significantly increased in mice with liver fibrosis housed under TN conditions, as shown in Figure 1D. A previous study reported that a deficiency in IL-10 resulted in abnormalities in the structure and function of BAT (21). We examined whether IL-10 could increase the severity liver fibrosis by inducing BAT dysfunction under thermoneutrality. Under TN conditions, the body weight of IL-10-deficient mice administered CCl_4_ was significantly lower than that of WT mice (Figure 5A). There was no significant difference in the fasting blood glucose level, which was checked before sacrifice, between the two groups (Figure 5B). At thermoneutrality, IL-10 KO mice exhibited severe liver injury. As shown in Figure 5C, serum AST, ALT, TCHO, and TG levels in IL-10 KO mice were much higher than in WT controls. By western blotting, the expression of α-SMA in the liver was also increased in mice lacking IL-10 (Figure 5D). As shown by histological analysis of the liver, collagen synthesis and accumulation was enhanced in the livers of IL-10 KO mice (Figure 5E). In addition, the expression of fibrotic genes was significantly increased in the livers of IL-10 KO mice (Figure 5F). However, there were no significant differences in the expression of genes related to BAT capacity between the two groups of mice (Figure 5G). Furthermore, no changes in the expression of pro-inflammatory cytokine such as *TNF-*α*, IL-1*β*, Mcp1*, and *IL-6* were found in BAT between IL-10 deficient mice and WT mice. A slightly larger in the size of lipid droplets was noticed in BAT sections from mice with IL-10 deletion. Taken together, at thermoneutrality, with the CCl_4_ induction, IL-10 KO mice displayed more severe hepatic damage than WT mice. However, further data were required to evaluate the BAT function in IL-10-deficient mice at thermoneutrality.

### Deletion of IL-10 Enhances Liver Immune Cell Infiltration at Thermoneutrality

**Figure 6:**
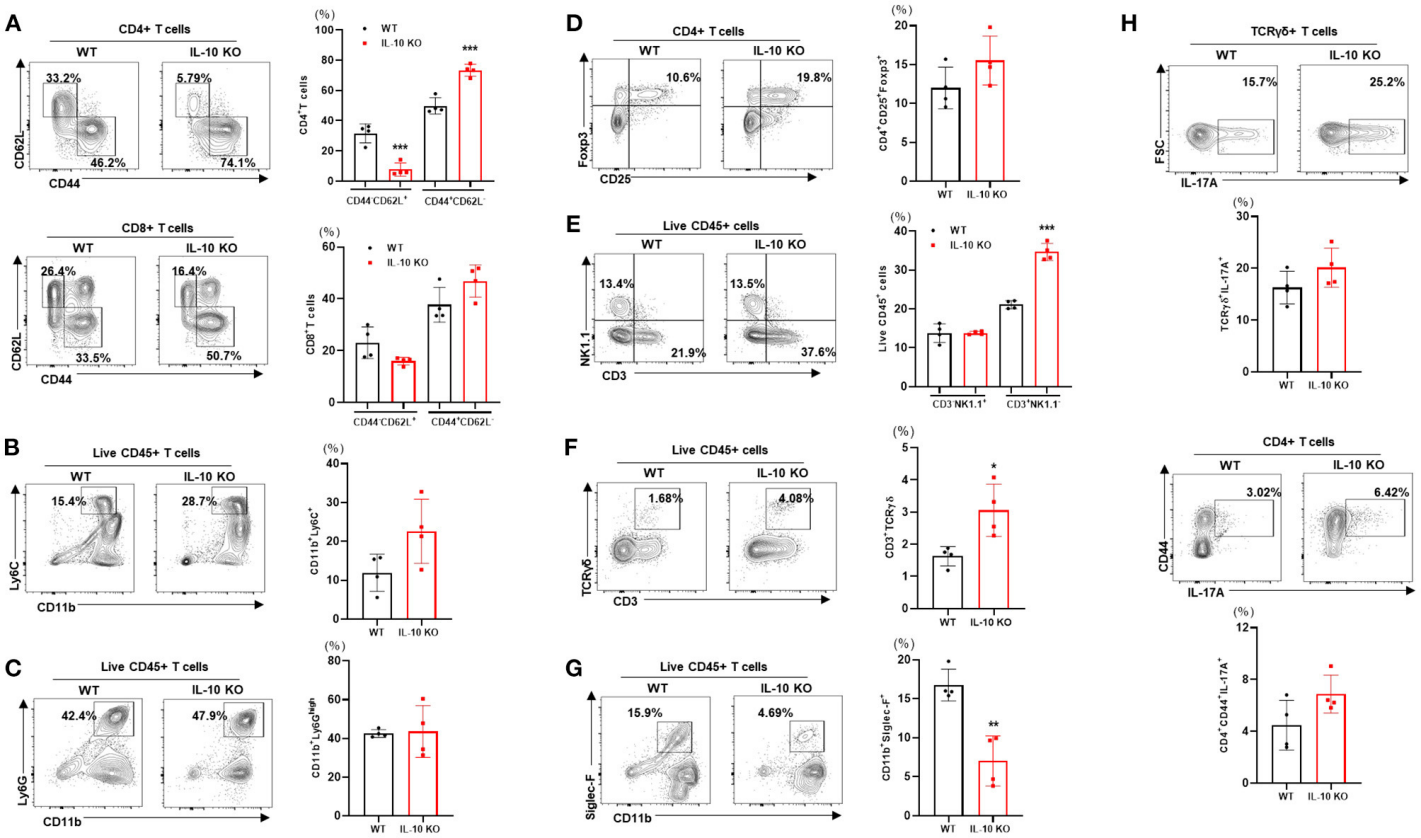
Deletion of IL-10 enhances immune cell infiltration of the liver at thermoneutrality. Isolated liver MNCs from control (*n* = 4) and IL-10 KO mice (*n* = 4) were analyzed for CD4+ T cells and CD8+ T cells **(A)**, monocytes **(B)**, eosinophils **(C)**, regulatory T cells **(D)**, γδ T cells **(E)**, neutrophils **(F)**, and NK cells **(G)** by flow cytometry. Liver MNCs were evaluated through intracellular staining for IL-17A-producing CD4+CD44+ T cells and γδ T cells **(H)**. Representative flow cytometry contour plots and statistical analyses of cell populations are presented. Statistically significant differences were determined by Student's *t-*tests. *p < 0.05, **p < 0.01, ***p < 0.001.

We examined the changes in liver-resident immune cells in WT and IL-10 KO mice under TN conditions. Compared with control mice, IL-10-deficient mice displayed a reduced proportion of naïve CD4+ T cells (CD62L+CD44-) cells in the liver. By contrast, there was an increase in the proportion of activated CD4+ T cells (CD62L-CD44+) (Figure 6A). However, no statistically significant changes in CD8+ T cells (Figure 6A), monocytes (CD11b+Ly6C+) (Figure 6B), neutrophils (CD11b+Ly6G^high^) (Figure 6C), Treg (CD4+CD25+Foxp3+) (Figure 6D), or NK cells (CD3-NK1.1+) (Figure 6E) were observed. However, IL-10 KO mice housed under TN conditions exhibited increased numbers of liver-infiltrating total T cells (CD3+NK1.1-) (Figure 6E) and γδ T cells (Figure 6F) compared with controls. Conversely, Siglec-F+CD11b+ eosinophils were reduced in the livers of IL-10-deficient mice with liver fibrosis (Figure 6G). A previous study by Y.P. Sharon Goh et al. indicated that eosinophils recruited to the liver can secrete IL-4, which triggers hepatocyte proliferation and liver regeneration (28). Thus, reduced recruitment of hepatic eosinophils may be one mechanism suppressing liver regrowth after injury in IL-10 KO mice. In addition, through intracellular staining, we did not find a significant difference in the numbers of IL-17A-producing γδ T cells or CD4+ T cells (Figure 6H) in the livers of IL-10 KO mice. Collectively, our findings demonstrated that deletion of IL-10 promoted liver inflammation but not a systemic inflammatory response.

### Conditioned Media From Differentiated Brown Adipocytes From IL-10 KO Mice Enhances HSCs Activation

**Figure 7:**
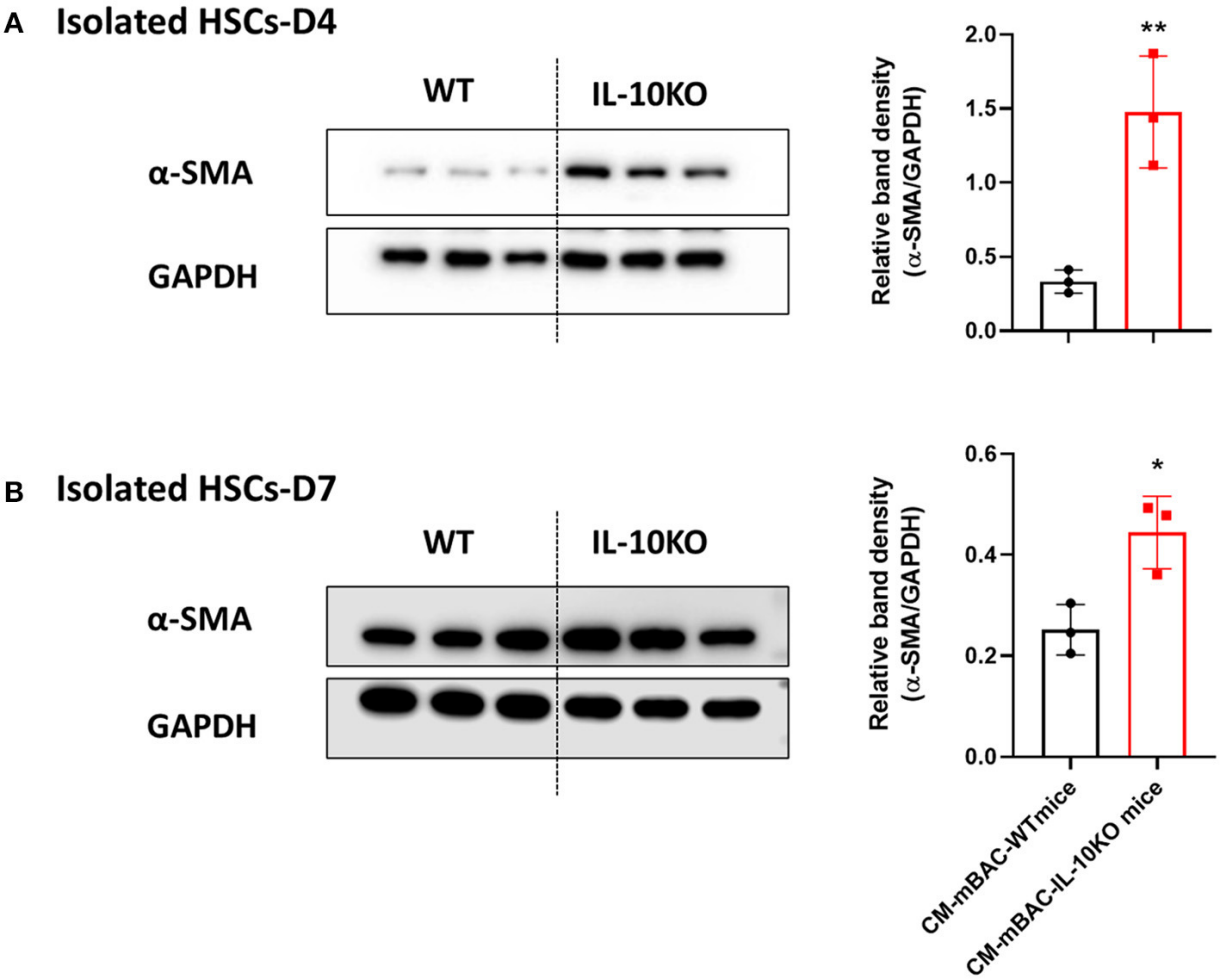
Conditioned media from IL-10-deficient brown adipocytes increased HSCs activation. CM-mBAC was collected after 24 h of culture of fully differentiated brown adipocytes. HSCs at day 4 **(A)** and day 7 **(B)** were cultured with CM-mBAC from WT and IL-10 KO mice. Statistically significant differences were determined by Student's *t-*tests. *p < 0.05, **p < 0.01.

As shown above (Figure 4), conditioned media from normal brown adipocytes could suppress the activation of HSCs. These findings demonstrated a beneficial role of BAT in hepatic fibrosis by directly altering HSCs. We next examined the effect of IL-10 deletion on the activation of HSCs by brown adipocytes. Primary brown adipocytes were isolated from WT mice and IL-10 KO mice and fully differentiated. Conditioned media was collected after 24 h of culture. Isolated HSCs at day 4 and day 7 were co-cultured with conditioned media from differentiated brown adipocytes from WT or IL-10 KO mice for 24 h. Conditioned media from differentiated brown adipocytes from IL-10 KO mice enhanced the expression of α-SMA in HSCs at day 4 and day 7 (Figures 7A,B). Thus, in contrast with WT brown adipocytes, IL-10-deficient brown adipocytes lost their ability to reduce HSC activation. These findings suggest a role for IL-10 in the regulation of HSC activation by brown adipocytes. Therefore, IL-10 may serve as a critical factor linking BAT and liver fibrosis.

## Discussion

Thermoneutrality induces severe inflammation not only in BAT but also in the liver, with a significant increase in the expression of genes associated with inflammatory and fibrotic responses. It is well-accepted that inflammation plays a key role in liver fibrosis progression (4). Of note, besides impaired BAT function (9), thermoneutrality can also induce intrahepatic immune cell infiltration. Several inflammatory infiltrated cells have been noticed following liver injury, specially, with liver fibrosis. It is well-known that inflammation plays a predominant role in hepatic fibrosis through the interaction between inflammatory cells, cytokines, and the related signaling pathways. Increased numbers of intrahepatic CD4+ and CD8+ T cells were observed in NAFLD patients with clinical and histological evidence of fibrosis, cirrhosis, and hepatocellular carcinoma (29). Zhisheng Her et al. suggested that CD4+ T cells play a key role in promoting hepatic fibrosis (29). Another study highlighted the abundance of activated CD8+ T cells in both human NASH (30) and a mouse model of high-fructose diet-induced liver fibrosis (31), and the abundance of NKT cells in non-alcoholic steatohepatitis triggered by a methionine- and choline-deficient diet (30). CD8+ T cells also contribute to the pro-fibrogenic activation of HSCs through STAT3 and depletion of CD8+T cells reduced NASH progression (32–35). Hepatic IL-17 producing cells are elevated in various experimental models of liver fibrosis, and mice deficient for IL-17 show resistance to liver fibrosis (26, 34, 36). It has been indicated that IL-17A has pro-fibrogenic effect (34, 36). IL-17A could stimulate Kupffer cells to express pro-inflammatory cytokines, and also, directly trigger HSC activation via the STAT3 signaling pathway (34). The population of IL-17A-producing cells was higher in livers of mice housed under TN compare to room temperature, as shown in our data. Therefore, TN could accelerate liver fibrosis in mice via IL-17A signaling pathway. While neutrophils are well-demonstrated to be recruited in severe liver inflammation and contribute to liver fibrosis progression, the role of Tregs in hepatic fibrosis is still controversial. Using FACS analysis, we found that thermoneutrality enhance the infiltration of most of pro-inflammatory immune cells, which promotes the activation of HSCs and hepatic fibrosis.

Furthermore, genetic deletion of IL-10 has been shown to increase systemic inflammation as well as to promote abnormal BAT function (21). In line with these findings, our data show that BAT dysfunction caused by TN housing and the deletion of IL-10 contributed to liver fibrosis progression.

Both the liver and the adipose tissue play many important roles in energy intake and utilization (37). Adipose tissue dysfunction can increase lipid flux to the liver (37). Furthermore, secreted factors are known to link BAT to other organs, including the liver (17). Crosstalk between BAT and the liver is critical for the development of fatty liver diseases (18). In that report, by culturing primary hepatocytes with conditioned media from BAT explants, the authors concluded that adiponectin, fibroblast growth factor 21 (FGF21), NRG4, and IL-6 play hepatoprotective roles (18). In our study, we focused on the link between BAT and liver fibrosis. Our data show that conditioned media from brown adipocytes also inhibits HSC activation and reduces α-SMA expression. In combination with previous findings, these data indicate that secreted factors from BAT have a protective effect not only on hepatocytes but also on HSCs. However, much is unknown about how the activation of HSCs is directly regulated by brown adipocytes. Changes in the function of BAT at thermoneutrality might have an impact on HSC activation. Additionally, inflamed BAT also could release various factors into the systemic circulation, exposing liver tissue to inflammatory cytokines that might accelerate liver damage (38).

Significantly elevated circulating levels of IL-10 were observed in mice housed under TN conditions after CCl_4_ challenge. Adipose tissue has been proposed to be a regulated source of IL-10 (39). A recent study demonstrated that IL-10 is expressed in macrophages derived from adipose tissue and its receptor is highly expressed in adipocytes (40). Further study is warranted on the impact of IL-10 produced by immune cells in adipose tissue under TN conditions on liver fibrosis. As an anti-inflammatory cytokine, under TN conditions, IL-10 is released to attenuate liver damage. In addition, another study reported that BAT mitochondria in IL-10-deficient mice have an abnormal structure and function (21). Thus, the lack of IL-10 induced brown adipocyte dysfunction, which exacerbated liver fibrosis in mice housed under thermoneutrality. Furthermore, deletion of IL-10 reversed the beneficial effect of conditioned media from dysfunctional brown adipocytes on HSC activation.

All of our findings suggest that BAT activity or with the presence of IL-10 could have beneficial effect on reduction of HSCs activation. Therefore, BAT function has been suggested to necessary for attenuating liver fibrosis. It has been demonstrated that lower Ucp1 expression in BAT of obese subjects compared to lean subjects (41). Thus, based on the association between obesity and lower BAT function, we can state that obese individuals may have a higher risk of liver fibrosis.

Our study has some limitations. First, we used only one mouse model of liver fibrosis. Using another animal model of hepatic fibrosis will be necessary to confirm the effect of TN on the liver. Second, we focused on HSCs in our *in vitro* studies to examine the impact of secreted factors from brown adipocytes on liver fibrosis. However, liver fibrosis is a complex process involving many liver cell types. The interactions among various types of liver cells might play an important role in regulating hepatotoxicity and BAT inactivation. Therefore, to investigate the detailed mechanisms linking BAT to the liver, brown adipocytes should be co-cultured with conditioned media from many others liver cell types, such as hepatocytes, Kupffer cells, and various liver-resident immune cells. Lastly, not only BAT but also white adipose tissue (WAT) is influenced by alterations in the temperature of housing conditions (23). Thermoneutrally housed mice also had more white fat and lower expression of Ucp1 and other thermogenic genes in both the WAT and BAT (23). TN may accelerate WAT inflammation by enhancing the recruitment of macrophages, neutrophils, CD4+ T cells, CD8+ T cells, and B cells (9). Thus, severe liver injury under TN conditions might be partially due to inflamed WAT.

In conclusion, this study demonstrated that BAT dysfunction caused by TN housing exacerbates liver fibrosis in mice. Deletion of IL-10 may further impair the function of BAT and boost pro-inflammatory responses in the liver. Conditioned media from WT brown adipocytes inhibited HSC activation, while conditioned media from IL-10-deficient brown adipocytes directly enhanced the activation of this cell type. Thus, IL-10 is an important factor mediating the protective role of brown adipocytes on HSC activation. Moreover, the dramatic increase in circulating IL-10 and its expression by the BAT in mice housed under TN conditions may be an adaptive response to attenuate liver fibrosis.

## Data Availability Statement

The raw data supporting the conclusions of this article will be made available by the authors, without undue reservation.

## Ethics Statement

The animal study was reviewed and approved by CNUH-019-A0071.

## Author Contributions

HTN conducted research, performed statistical analysis, and wrote the manuscript. JSM, JWT, HYL, S-HK, and Y-SL analyzed the data and provided constructive comments. J-HJ provided IL-10 KO mice. H-SY designed research, wrote manuscript, and supervised the study. All authors contributed to the article and approved the submitted version.

## Conflict of Interest

The authors declare that the research was conducted in the absence of any commercial or financial relationships that could be construed as a potential conflict of interest.
